# Flora richness of a military area: discovery of a remarkable station of *Serapiasneglecta* in Corsica

**DOI:** 10.3897/BDJ.10.e76375

**Published:** 2022-02-24

**Authors:** Margaux Julien, Bertrand Schatz, Simon Contant, Gérard Filippi

**Affiliations:** 1 CEFE, CNRS, Univ Montpellier, EPHE, IRD, Montpellier, France CEFE, CNRS, Univ Montpellier, EPHE, IRD Montpellier France; 2 Ecotonia, 60 Rue Tourmaline, Eguilles, France Ecotonia, 60 Rue Tourmaline Eguilles France; 3 Independant Botanist, 7 Chemin de Bourdaric, Bourdaric, France Independant Botanist, 7 Chemin de Bourdaric Bourdaric France

**Keywords:** plant biodiversity, *Serapiasneglecta*, military area, Corsica, conservation

## Abstract

One of the central issues in conservation today is identifying areas rich in biodiversity for priority conservation. On a global scale, the Mediterranean area is a biodiversity hotspot and, locally, Corsica contains high biodiversity with interesting sites for conservation. An inventory of flora was undertaken on the Solenzara military airbase. Five hundred and fifty-two plant species were inventoried, which represent an important species richness. Amongst these species, certain are rare or endemic. A large population of Serapiasneglectasubsp.neglecta was found and the size of this population was estimated. This species is localised at a global scale and has a protection status. This is the largest population known, with more than 155,000 individuals on the 550 ha of the airbase. Nineteen plant species have national protection status and 15 are classified as invasive alien species. The Solenzara airbase has a role in conserving many species; a management plan would be appropriate.

## Introduction

*In situ* conservation can take different forms: at the species level, it is possible to select the most vulnerable species and develop conservation programmes to improve the conservation status of these target species (Guyot and Muracciole 1995, Fenu et al. 2020, Fišer et al. 2021). However, it is impossible to conserve all plant species through specific programmes, especially for funding and logistical reasons. Another approach is to focus conservation efforts on priority areas and preserve the whole community present in these areas (Coates and Atkins 2001, Bonn and Gaston 2005, Fenu et al. 2010, Zhang et al. 2017). This can be done by creating protected areas with varying degrees of constraints to conserve the present biodiversity. These two approaches are perfectly compatible and complementary in practice: conservation plans targeting certain species can be implemented in protected areas (Heywood et al. 2018).

On a global scale, biodiversity hotspots have been defined as areas where biodiversity is significant, generally with a high level of species richness and endemism (Myers et al. 2000). One of the priority areas defined by Myers is the Mediterranean Basin. The many islands in the Mediterranean Basin have allowed high endemism due to their geographical isolation (Thompson et al. 2005). It is, therefore, relevant to look for priority areas for conservation within these hotspots, which could be defined as hotspots within hotspots (Cañadas et al. 2014).

Corsica is an island of 8748 km² situated in the Mediterranean Basin. This Island is a flora refugia and a biodiversity hotspot, due to its location and history (Medail and Quezel 1997, Médail and Diadema 2009, Lestienne et al. 2019). The Corsican landscapes vary with massifs exceeding 2300 m of altitude (Jeanmonod and Gamisans 2007) with certain hotspots (Vogt-Schilb et al. 2016, Schatz 2017). The Corsican flora is well-known notably thanks to Jeanmonod and Gamisans (2007) and the various analyses carried out from this flora (Jeanmonod et al. 2009, Schlüssel et al. 2014, Jeanmonod et al. 2015). Corsican flora is composed of 2238 indigenous taxa, with 302 sub-endemic taxa. It faces certain threats, like land planning, pastoral abandonment or the introduction of invasive species. It is, thus, necessary to protect this rich flora (Jeanmonod and Gamisans 2007).

We are interested in a military base in Corsica. Indeed, military bases are important areas for biodiversity because they are closed to the public, are not heavily impacted and these areas have soils that are often poorly fertilised and untreated due to old installations, so they often have high biodiversity (Warren et al. 2007, Seitre 2017, Massó et al. 2019). Military bases represent a significant part of the surface of the Earth (1-6%) (Zentelis and Lindenmayer 2015). However, they are military training areas with regular disturbances, which finally create a mosaic of habitats and the large surface areas allow for spacing of disturbances. Pioneer species and later species in the plant formations can settle and persist in these conditions (Warren et al. 2007). Therefore, they are often areas of high plant biodiversity and can play an essential role in the conservation of flora. To this end, it is necessary to know what is at stake in these areas, which are not accessible to the public. Knowledge of the issues at stake makes it possible to suggest favourable management and enhance the populations of rare or threatened species. Furthermore, it is necessary to allow military activities to co-exist with conservation activities, leading to conflicts (Lee Jenni et al. 2012).

Our main goals are: 1) to elaborate a checklist of vascular flora in the airbase of Solenzara to better assess the floristic richness and the conservation priorities of this area; and 2) to quantify the considerable population of *Serapiasneglecta* De Not., which is a protected sub-endemic species of orchid, highly remarkable on the airbase.

Rich plant biodiversity is expected due to the geographical location and use of the area. The list of species in the Solenzara military zone contributes to the knowledge of the flora in this sector, to which access is restricted and regulated, making it possible to understand better and locate the challenges of this zone and best conserve the remarkable species.

## Material and methods

### Study site and local context

Our study area is localised in the Mediterranean Basin, one of the 25 biodiversity hotspots (Myers et al. 2000), where the flora is diversified, with a high level of endemism (Médail and Verlaque 1997). Moreover, the Mediterranean Basin is composed of several islands. Due to their insular nature, these islands contain much of the species richness of the Mediterranean area (Kier et al. 2009, Médail and Diadema 2009, Schatz 2017). Flora richness in Corsica, where our study area occurs, is higher than in metropolitan France (0.29 taxon per km² in Corsica and 0.09 taxon per km² in France) and endemism is important, with 5.9% strictly endemic plants and 13.5% sub-endemics (Gamisans and Jeanmonod 1995).

**Figure 1.**
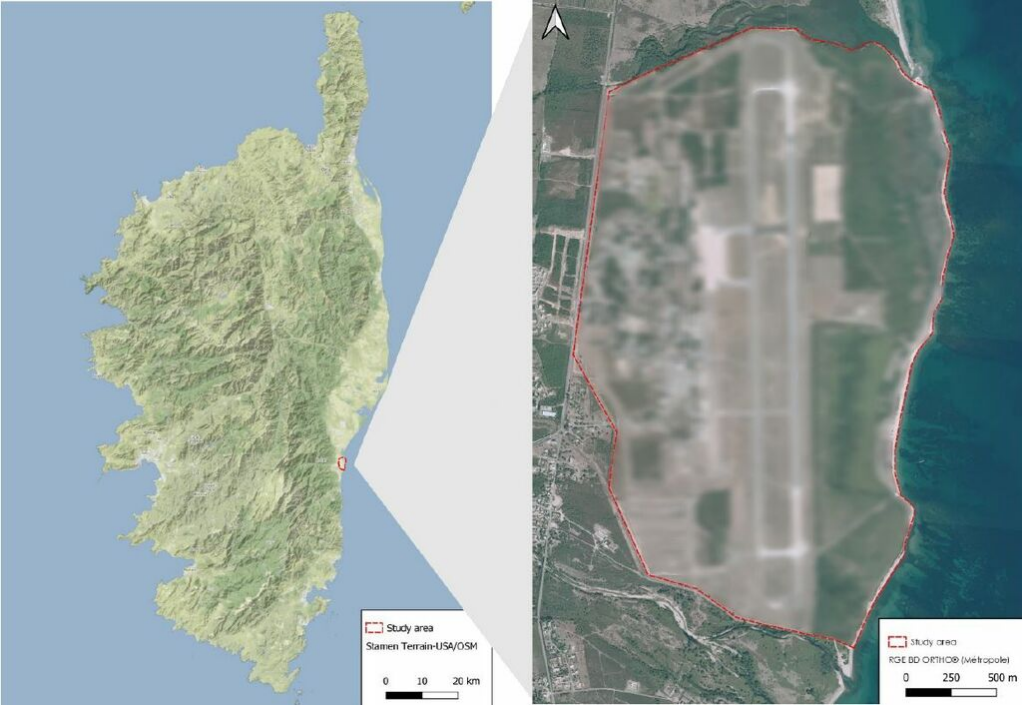
Study site and localisation in Corsica.

Our study site is the airbase called n°126 Solenzara, a French Air Force base, located at Ventiseri (Fig. 1) and was created in 1952 by the North Atlantic Treaty Organisation (NATO) (Giannini 2019). NATO chose this area because it is airspace with little traffic and marshy areas are suitable for test firing. Major military development work was undertaken after 1956, including the drying out of wetlands. For the airbase, the draining of these wetlands appears to have benefited from a significant input of topsoil from major road infrastructure works carried out before 1973 (Bonfond 2018, Giannini 2019).

This airbase is about 2 km wide (east-west axis) and about 3.3 km long (north-south axis) and it is located approximately 40 km north of Porto-Vecchio, on the east coast of Corsica. The Palo Pond (Ramsar site) surrounds the airbase in the north, the Travo River in the south, a dune site and the Mediterranean Sea in the east and a national road and an urbanised area to the west. The inventory takes place in the entire airbase, including the wetland area east of the beach (this second part corresponding to a site protected by the Conservatoire du Littoral). The total area surveyed is about 550 ha. This study area is a military site, which implies that it is closed to the public. It is also an airport area. Airports are interesting for biodiversity; lawns around strips are particularly favourable to orchids (Seitre 2017). Indeed, most runways are bordered by open grasslands, managed by regular mowing for aeronautical safety reasons.

There are three main parts to the airbase. The westernmost part consists of buildings and low grasslands, with some woodland. In the central area, the landing strips are surrounded by low vegetation, regularly maintained by mowing. Finally, the easternmost part consists of wetlands and scrub. A de-sodding exercise was carried out in 2019 in this third part. The airbase is located in the coastal and thermomediterranean belts. These belts are rich in plant species, particularly the thermomediterranean belt (Schlüssel et al. 2014).

### Sampling method

Several floristic inventories have been carried out and are compiled here to give an almost exhaustive list of the flora present on the airbase. Since 2017, the Ecotonia Consultancy has regularly intervened on the airbase to carry out floristic inventories. From 2017 to 2019, the inventories focused on the central area, around the runways and the northeast wetland (These inventories took place in March, April, May and October). In 2020, a complete inventory of the wetland was conducted (March to June). In 2021, an inventory took place in the northwest and another in the southwest of the airbase (March to June). This completed the data acquired since 2017.

Then, we added floristic data acquired by the Corsican National Botanical Conservatory (CBNC), following inventories conducted in 2018 and 2019 (May to July) (Delage and Hugot 2019). These data complement the inventories of Ecotonia, covering a large part of the airbase and especially along the sea.

More targeted inventories have been previously conducted: in 2010 (April) by the Association of Friends of the Corsican Regional Natural Park on areas around Palo Pond, focused on remarkable flora (Massoni et al. 2010) and, in May 2012 and April 2017, by the Cyrno-Mediterranean Orchidology Association (ACMO), focused on Orchidaceae (Denis Allemand, com. pers.).

The objective of the inventories was to conduct a systematic sampling to determine all species present in the study area. This allowed us to determine a precise list of taxa present. We then compared this list to the protected and threatened species list (decree of 20 January 1982, modified by the decree of 31 August 1995; decree of 24 June 1986 in French law) (Delage and Hugot 2015, UICN France et al. 2018). We noted the degrees of endemism and rarity for each species given by the Flora Corsica determination key (Jeanmonod and Gamisans 2007, Jeanmonod et al. 2009). For several species, the subspecies is not specified in the list we have established. We have chosen to take the lowest degrees of rarity in the Flora Corsica determination key: we likely contacted the most common subspecies. We have taken the corresponding degree of endemism. To find out the proportion of endemic species, we removed non-native species. Finally, we compared the list with the invasive alien species list of Corsica (Petit and Hugot 2019).

### Estimation of the density of *Serapiasneglecta*

**Figure 2.**
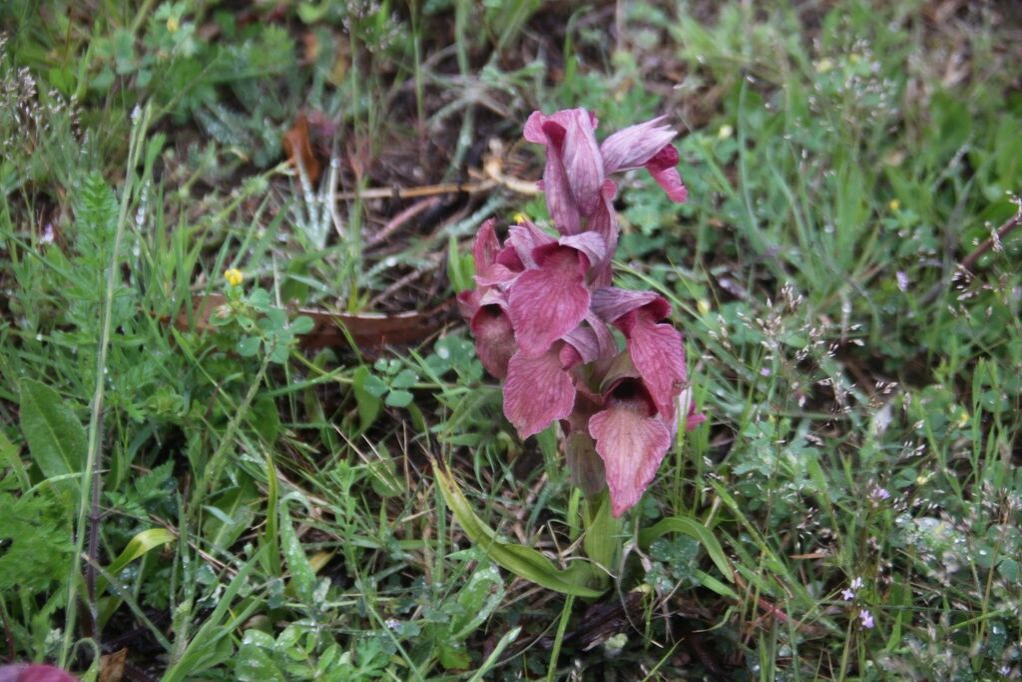
*Serapiasneglecta* on Solenzara airbase (April 2021).

Amongst inventoried species, we found a large population of *Serapiasneglecta*, a nationally protected species of orchid (Fig. 2). Its range is limited to the extreme south-east of mainland France, Corsica (mainly around the south coast) and Sardinia, Sicily, the extreme south-east of Italy and along the east coast of the Adriatic (Croatia, Albania and Greece) (Delforge 2016). We estimated the size of the population. Firstly, we defined by cartography using the software QGIS the favourable areas for *S.neglecta*. We defined five large homogeneous zones:

- The edges of runways: the vegetation is regularly mowed; the environment is very favourable to *Serapias*.
- Wetlands: *Serapias* grow in smaller quantities, but are present.
- Lawns near buildings: these lawns are regularly maintained. There is little competition and *Serapias* are numerous.
- The south-western zone: vegetation is high. *Serapias* are present in a few places, but in reduced quantity.
- The north-western zone: *Serapias* are present, but scattered. The vegetation is closing in.

We chose a representative area of at least 600 m² in each homogeneous zone, where we made an exhaustive count. We then extrapolated the number of *S.neglecta* per area.

## Results

### Flora composition

**Figure 3.**
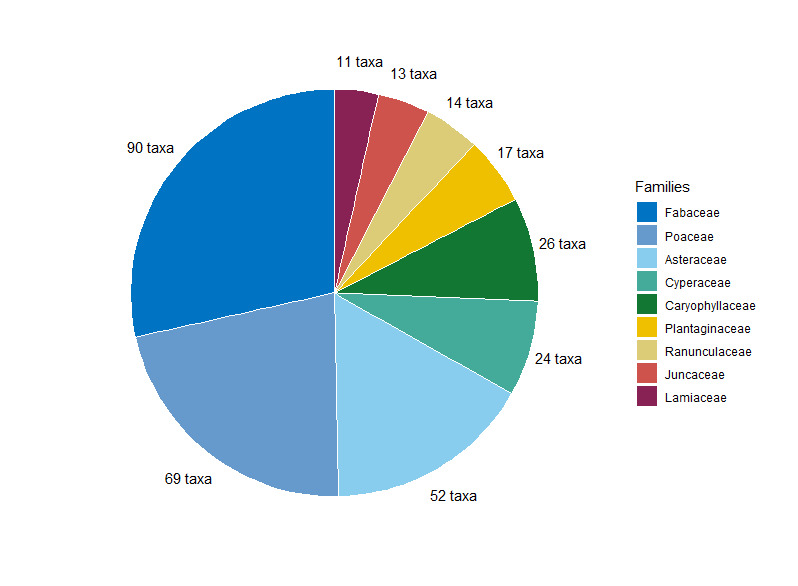
Distribution of the most contacted families on the airbase.

The 552 taxa found in the study area belong to 279 genera and 74 plant families (Suppl. material 1). Amongst these taxa, there are 45 sub-species, 503 species, four varieties and one hybrid. The families with the most species are Fabaceae (90 species), Poaceae (69 species) and Asteraceae (52 species) (Fig. 3). The genera with the most species are *Trifolium* (28 species), *Carex* (13 species), *Juncus* (13 species) and *Vicia* (13 species).

Only 2.5% of species are sub-endemic and none is strictly endemic. Many protected species are present on the base and 19 species benefit from national protection, representing 3.4% of the plant taxa on the airbase. There are also 15 invasive alien species, representing 2.7% of the taxa present.

**Figure 4.**
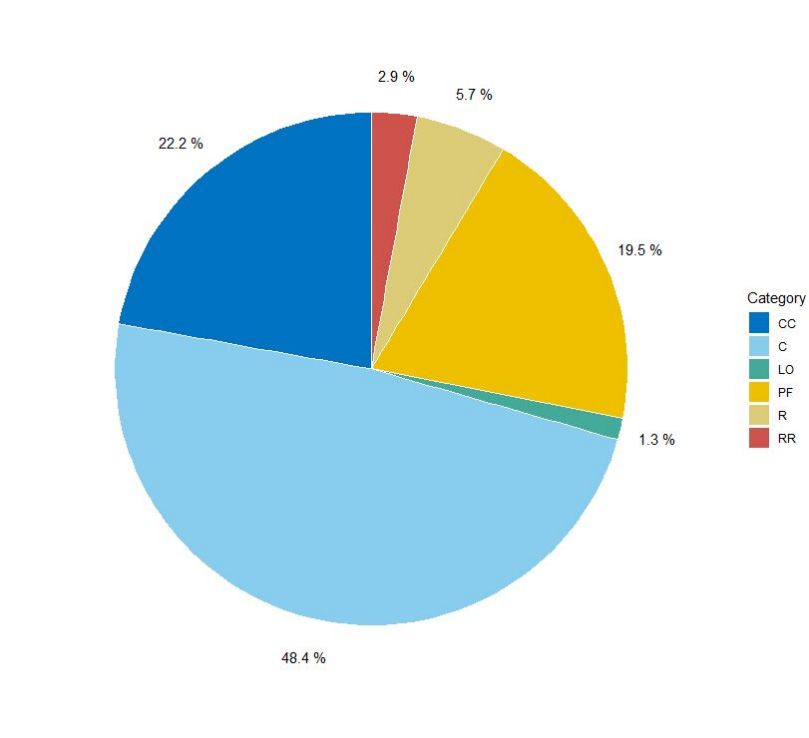
Proportion of taxa by rarity category. CC: Very Common, C: Common, LO: Localised, PF: Uncommon, R: Rare, RR: Very Rare.

More than 70% of the species in the database are common (C) or very common (CC) (Fig. 4). Only 8.6% are rare (R) or very rare (RR).

### Presence of a remarkable population of *Serapiasneglecta*

A large part of the base is favourable to *S.neglecta*: the population extends on both sides of the runways and the lawns near the buildings and wetlands. There are also several other orchid species: *S.cordigera* L., *S.parviflora* Parl., *S.lingua* L., *Anacamptismorio* (L.) R.M.Bateman, Pridgeon & M.W.Chase, *A.laxiflora* (Lam.) R.M.Bateman, Pridgeon & M.W.Chase and *A.papilionacea* (L.) R.M.Bateman, Pridgeon & M.W.Chase. However, the case of *S.neglecta* is particularly remarkable because this species benefits from a national protection status and it is a sub-endemic species with a very localised distribution worldwide. This species is classified as near threatened on the World and European Red Lists of the IUCN (Rankou 2011). In Corsica, this species is present and often abundant. However, no other population of comparable size is known. Note here the rather shallow tuber depth of the orchids present in this area (on average 4.33 cm in the base vs. 7.36 cm outside the base) due to the shallow soil on a pebble bed (due to ancient variations in the course of the Travo River Delta) (see more information in Suppl. material 2).

Concerning the risks of confusion with related species, we confirm the presence of Serapiasneglectasubsp.neglecta De Not. on our study site and not that of Serapiasneglectasubsp.apulica Landwehr which is rather a subspecies of *Serapiasorientalis* (Greuter) H.Baumann & Künkele (Lorenz 2001, Delforge 2016), located in the Apulian coast in Italy, with a more compact inflorescence with fewer flowers, a more lanceolate epichile and an earlier flowering (Delforge 2016). Similarly, we also confirm the presence of Serapiaslinguasubsp.lingua L. on our study site and not that of Serapiaslinguasubsp.tunetana B.Baumann & H.Baumann which is only present in Tunisia, with a lower number of flowers, a shorter, less wide and more lanceolate epichile, a lighter colour and a later flowering (El Mokni and Domina 2019).

The estimated area favourable to *S.neglecta* is 160 ha on the entire airbase. We assessed the density on different locations: 0.12 *S.neglecta*/m² on the edge of the runways, 0.009 *S.neglecta*/m² in the wetlands, 0.2 *S.neglecta*/m² on the lawns near the buildings, 0.007 *S.neglecta*/m² in the south-western zone and 0.05 *S.neglecta*/m² in the north-western zone. Extrapolating, we estimate that the population of *S.neglecta* is at least 155,000 individuals on the entire airbase.

This estimate is probably underestimated due to the nature of the species. Indeed, the individuals do not flower every year. We, therefore, see only a fraction of the population present. Depending on the year, densities of *Serapias* have been very variable. In 2019, the winter was arid (especially during the previous winter and spring), which was not favourable to orchids; during this year, we only observed hundreds of *Serapias*. Almost no individuals were visible in the spring on the airbase. Between 2020 and 2021, we compared the density of an area particularly rich in *Serapias* of about 900 m². The number of individuals was 2.5 times higher in 2021 than in 2020 (0.28 *S.neglecta*/m² in 2020 versus 0.81 in 2021).

### Other remarkable species

Some species are remarkable for their status: for example, *Gratiolaofficinalis* L. is classified as vulnerable (VU). There are 13 species classified as near threatened (NT). *Ranunculusrevelierei* Boreau have national protection and is NT on theCorsican IUCN Red List. This species is also sub-endemic and rare in Corsica. Four nationally-protected species and very rare in Corsica are found: *Trifoliumcernuum* Brot., *Gratiolaofficinalis* L., *Ranunculuslingua* L. and *Anemonecoronaria* L. Finally, *Salixapennina* A.K.Skvortsov is very rare and *Serapiasolbia* Verg., Saginasubulatavar.gracilis Foucaud & Simon and *Ranunculusrevelierei* are rare. These three species are also sub-endemic.

One subspecies is to be considered as potential on the airbase; Bromushordeaceussubsp.thominei (Hardouin) Braun-Blanq. This subspecies is present in Corsica, but challenging to determine. We consider it as potential, but it has not been taken into account during the analyses.

*Saginasubulata* (Sw.) C.Presl belongs to the huge family of Caryophyllaceae. Two subspecies exists: S.subulatasubsp.revelierei (Jord. & Fourr.) Rouy & Foucaud, an endemic Corso-Sardinian orophyte and S.subulatasubsp.subulata (Sw.) C.Presl, a southern and western European species. The latter is currently divided into two varieties, one of which is present throughout its range (var. subulata (Sw.) C.Presl), but the other is not known: var. gracilis Foucaud & Simon. It is only reported in France (Provence and Corsica), although it is potentially present elsewhere (Sardinia, Tuscany, Liguria, Spain). It is found in a singular environment: shallow temporary ponds with siliceous substrates in the Mediterranean climate. This environment, which is highly stressful for a plant, has led this taxon to adopt a therophyte biological type – its life is limited to a few weeks or months, whereas var. subulata is hemicryptophytic. This adaptation is typical of Mediterranean environments and seems to be an important evolutionary event that may justify this variety being treated at a higher taxonomic rank soon. If specific research is carried out, it could one day lead to the description of a new high-ranking taxon (species or subspecies), whose worldwide distribution area would at best be restricted, or even endemic, to the French Mediterranean region. In Solenzara, this tiny plant has found unusual secondary habitats: the ruts made by vehicles driving on the base. Other annual species accompany it with similar ecologies, some of which are rare: *Ranunculusrevelierei*, *Lotusconimbricensis* Brot., *Solenopsislaurentia* (L.) C.Presl, *Lysimachiaminima* (L.) U.Manns & Anderb. and *Lythrumportula* (L.) D.A.Webb.

## Discussion

The Solenzara airbase is rich in plant species. Its location explains this richness since Mediterranean islands have high biodiversity (Medail and Quezel 1997, Kier et al. 2009, Médail and Diadema 2009). It is also explained by the nature of the area since military areas often have a high level of species richness (Warren et al. 2007, Zentelis and Lindenmayer 2015, Massó et al. 2019), but also because this is an airbase: airports generally harbour a high level of biodiversity, particularly of orchids (Seitre 2017). Airports are usually mown regularly for safety reasons related to aircraft traffic; this mowing, especially near runways, favours species with little competition, such as orchids. It is, therefore, not surprising to find protected and remarkable species amongst this richness. We have shown that 23.2% of the indigenous taxa present in Corsica are present on the military base of Solenzara, on only 550 ha. In comparison, 688 species were inventoried on the 16,175 ha of the Hohenfels training area in Germany, which represents 27% of the species richness of Bavaria (Warren et al. 2007). This military area is almost 30 times larger than the Solenzara airbase.

There are 302 sub-endemic taxa, 688 rare and very rare taxa, 191 taxa with a protection status and 64 invasive species in Corsica (Jeanmonod and Gamisans 2007, Jeanmonod et al. 2009, Petit and Hugot 2019). This represents a significant proportion of these categories, given the small surface area of the airbase. Thus, 6.5% of the rare taxa, 4.3% of the sub-endemic taxa (9.8% if we consider only the 133 taxa of the littoral and thermo-Mediterranean belts) and 10.0% of the protected taxa of Corsica are present on the airbase. On the other hand, 23.4% of the invasive species of Corsica are present on the airbase.

To a large extent, this sector represents a control zone for the flora of the eastern coast of Corsica before urbanisation, which is locally very close. However, this base was created on the former delta area of the Travo River, which explains the shallow soil on a pebble bed. This particular situation probably limits the presence of several other species and makes the diversity of this site even more exceptional.

Proportions of endemic and rare species are not negligible; moreover, we have noted the presence of species to be conserved as a priority. We presented the exceptionally abundant population of *Serapiasneglecta*, which is the largest known to date. More generally, orchids are present in all areas maintained by mowing in the airbase (12 different species of orchids). Other species as *Ranunculusrevelierei* are also remarkable and deserve better consideration.

Military activities (napalm use, nuclear weapons, conflicts) are often considered as threats to biodiversity (Lawrence et al. 2015, Massó et al. 2019). However, despite these negative impacts, military areas can be an opportunity for biodiversity conservation. It is not always easy because, for the military, it is not acceptable to restrict the training of soldiers because of environmental constraints (Lee Jenni et al. 2012). The airbase has a role to play in the conservation of these species. On this military base, training areas are small and localised and there are still many natural areas. It is, therefore, entirely possible to set up conservation actions for remarkable species. Currently, the management of the base allows orchid populations to extend. However, improvements are possible and there are areas where management can be targeted to benefit these species. A management plan that promotes plant biodiversity on the airbase, considering the issues, could further increase the richness and perhaps promote rare or endemic species present near the airbase. There is also a need for a management plan to control invasive species.

The Solenzara airbase can be considered as a biodiversity reserve; there is little human activity, some areas being very infrequently visited (Médail and Verlaque 1997). This is a real opportunity for plant conservation. This richness gives the military base a responsibility for conserving this natural heritage; this situation should be recognised by a protection status for this site used in France (such as APHN: Decree of natural habitat protection or APPB: Decree prefectoral of biotope protection).

## Supplementary Material

F0D04EB5-D3EF-5CF5-8400-A722C045EBE5 10.3897/BDJ.10.e76375.suppl1 Supplementary material 1 Complete list of the flora of Solenzara Data type table File: oo_640208.xlsx https://binary.pensoft.net/file/640208 Julien, M; Schatz, B; Contant, S; Filippi, G

66F1E68B-0F1E-5A3E-B740-14947F9E89BA 10.3897/BDJ.10.e76375.suppl2 Supplementary material 2 Depth of root system of several *Serapias* species Data type figure and comments File: oo_596040.docx https://binary.pensoft.net/file/596040 Julien, M; Schatz, B; Contant, S; Filippi, G
