# Supplementary material for: Flora richness of a military area: discovery of a remarkable station of Serapiasneglecta in Corsica
Source: Biodivers Data J. 2022 Feb 24;10:e76375. doi: 10.3897/BDJ.10.e76375 (PMC8894324; doi:10.3897/BDJ.10.e76375)
Supplement: Supplementary material 2 — Depth of root system of several Serapias species [file bdj-10-e76375-s002.docx]

During the inventories carried out in 2019, some orchids had been unearthed by boars that consume the tubers. This damage concerned individuals of *Serapias neglecta*, *S. lingua* and *Anacamptis papilionacea*. On this occasion, measurements of the root system were carried out on the excavated individuals. Outside the airbase, the same measurements were made on other individuals, also excavated by boars.

For each species, we performed comparisons of root depth means using the student test, after checking the conditions of application. Means were different for all species, with smaller root systems on the airbase: for *S. neglecta*, the root systems measured on average 4,33 cm on the airbase and 7,36 cm outside the airbase (t = 15; df = 59; p-value < 2^e^-16); for *S. lingua*, on average 3,90 cm on the airbase and 7,36 cm outside (t = 20; df = 38; p-value < 2^e^-16), and for *A. papilionacea*, on average 4,03 cm inside and 6,78 cm outside (t = 15; df = 46; p-value < 2^e^-16).

Therefore, the difference in soil depth is quite marked: in the airbase, the soil is shallow due to the site history.


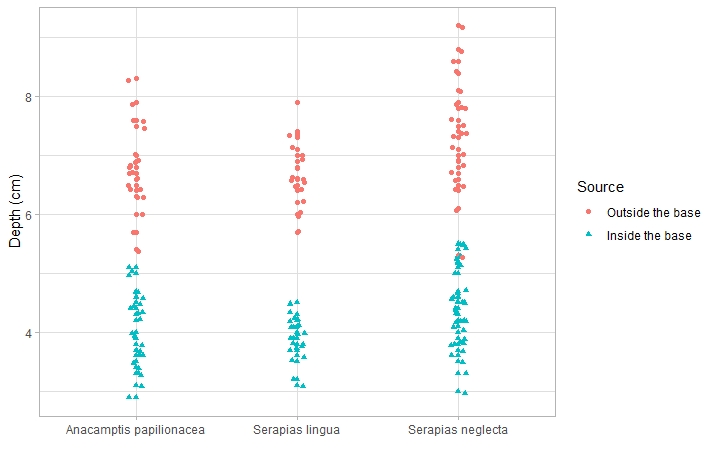


Supplementary figure: Mean depth of root system
